# Case report: Resection of a giant right ventricular myxoma

**DOI:** 10.3389/fsurg.2023.1140016

**Published:** 2023-02-27

**Authors:** Jin Rao, Qian Yang, Liang Yin, Yue Yu, Wang Xi, Jibin Xu, Yufeng Zhang, Zhinong Wang

**Affiliations:** Department of Cardiothoracic Surgery, Changzheng Hospital, Naval Medical University, Shanghai, China

**Keywords:** ventricular myxoma, resection, right ventricle outflow tract, echocardiography, cardiac computed tomographic angiography

## Abstract

Myxoma constitutes the main subtype of all benign cardiac tumors, tending to be more common in women and occurring mostly in the left and right atria. Its classic clinical presentations are intracardiac obstruction, embolization, and systemic or constitutional symptoms, such as fever, in decreasing order. Several imaging techniques such as echocardiography, computed tomography, and angiocardiography contribute to the diagnosis of myxoma, ruling out significant coronary diseases, and assessment of myocardial invasion and tumor involvement of adjacent structures. Surgical resection is the only effective therapeutic option for patients with cardiac myxoma. Here, we report a unique case of a middle-aged man who presented with a giant myxoma and a 3-day history of chest tightness and shortness of breath after physical activity. Subsequently, transthoracic echocardiography revealed a mass of solid echodensity located within the right ventricle, complicated by abnormal hemodynamics. A cardiac computed tomographic angiography showed a large homogeneous density filling defect consuming most parts of the right ventricle and protruding from beat to beat. A surgical resection and histological study later successfully confirmed the diagnosis, and the patient's postoperative recovery course was found to be uneventful.

## Introduction

Primary cardiac tumors are uncommonly encountered clinical problems, of which myxomas account for over 50% and 10% of cardiac tumors in adults and children, respectively (1, 2). The vast majority of myxoma cases are sporadic and occur more commonly in women (3). Only less than 5% of myxomas, which have varying sizes, occur in the ventricles (4). Our search for myxoma cases for the year 2021 in the PubMed database revealed that 120 of all 126 myxomas occurred in the atria, having a size of 1–10 cm. In this article, we describe a patient with a 2-month history of increasing chest tightness and shortness of breath and a significant distention of jugular veins. Both transthoracic echocardiography (TTE) and cardiac computed tomographic angiography (CCTA) revealed a space-occupying mass in the ventricle. Histopathology confirmed the diagnosis of myxoma.

## Case presentation

A 53-year-old man presented to the cardiovascular surgery clinic on 14 September 2021, with the chief complaint of a 3-day history of chest tightness and shortness of breath. The above-mentioned symptoms began to appear 3 days before presentation, after physical activity, with each episode lasting 5 min and resolving after a rest. The patient immediately visited the local hospital, where relevant tests were performed, which suggested an intraventricular space-occupying lesion, mild tricuspid regurgitation, incomplete right bundle branch block, and ST/T changes. Since a radical cure was unavailable in the previous healthcare setting, he was transferred to our hospital for surgical treatment. The patient had a medical history of hypertension for more than 3 years, for which he was not taking any pressure-lowering medications, and had undergone one surgical procedure for a right-leg fracture over two decades ago.

**Figure 1:**
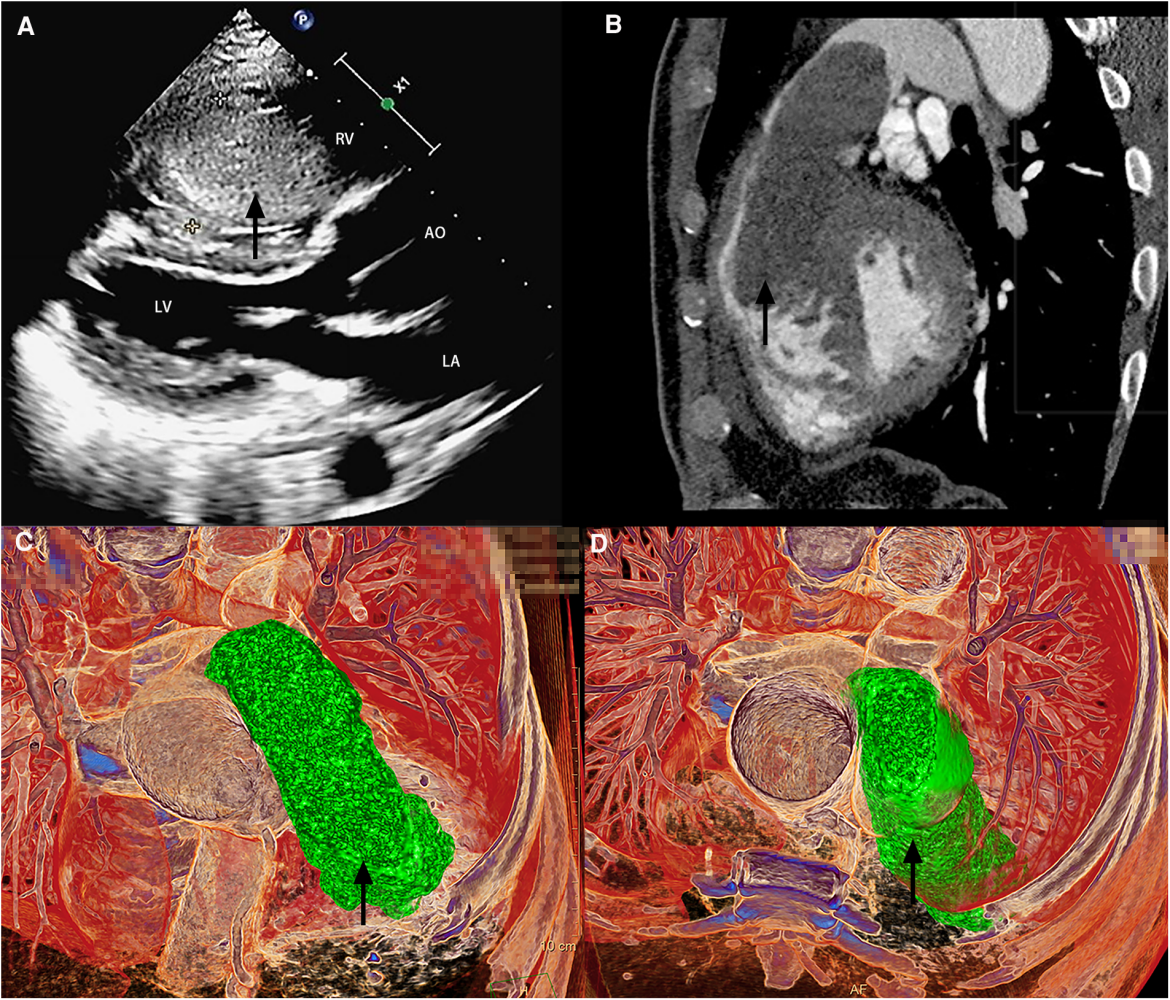
Imaging examination. Transthoracic echocardiography (**A**) and cardiac computed tomographic angiography (**B–D**) revealed a mass located within the right ventricle (black arrow). RV, right ventricle; LA, left atrium; LV, left ventricle; and AO, aorta.

On hospital admission, his blood pressure level was 146/90 mm Hg, and body temperature, pulse, and respiratory rates were within normal limits. On physical examination, a significant distention of the jugular veins and a systolic blowing murmur were noted on the 4th and 5th intercostal spaces on the left margin of the sternum. Cardiac enlargement toward the lower left was recorded on percussion. No abnormalities were detected on respiratory auscultation. TTE revealed a mass located within the right ventricle (Figure 1A and Supplementary Video), complicated by moderate tricuspid regurgitation and ascending aortic dilatation with mild aortic valve regurgitation. Chest CT and CCTA showed a large homogeneous density mass consuming most parts of the right ventricle (RV) with protrusion into the RV outflow tract and proximal main pulmonary artery during systole (Figures 1B–D) and relocation during diastole. According to the evaluation of TTE and CCTA, the possibility of multiple myxomas in other cardiac chambers was minimal.

**Figure 2:**
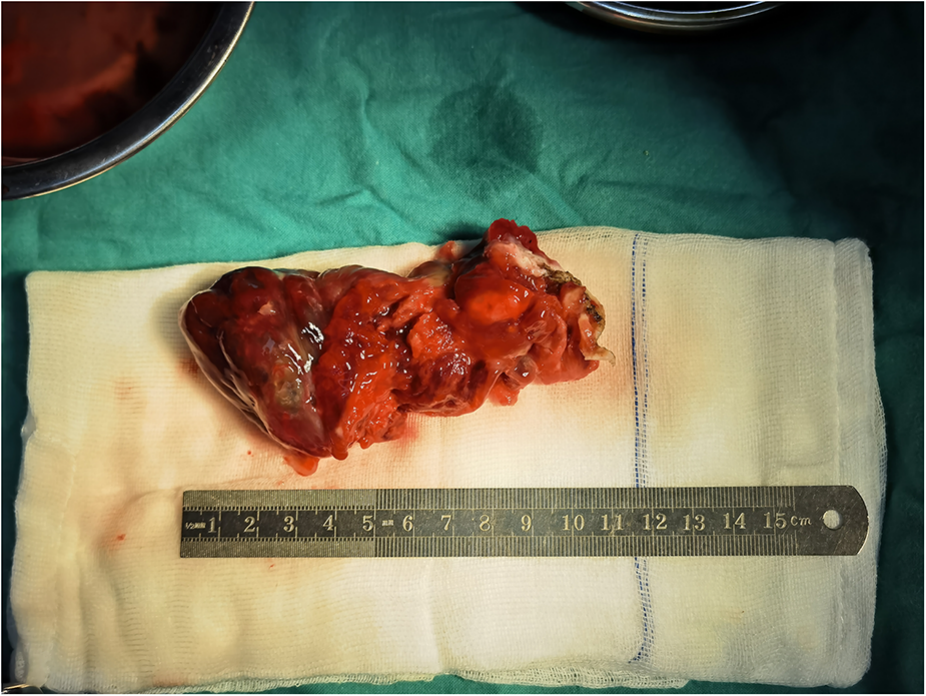
The patient underwent a resection of the mass (measuring 110 × 51 × 43 mm).

**Figure 3:**
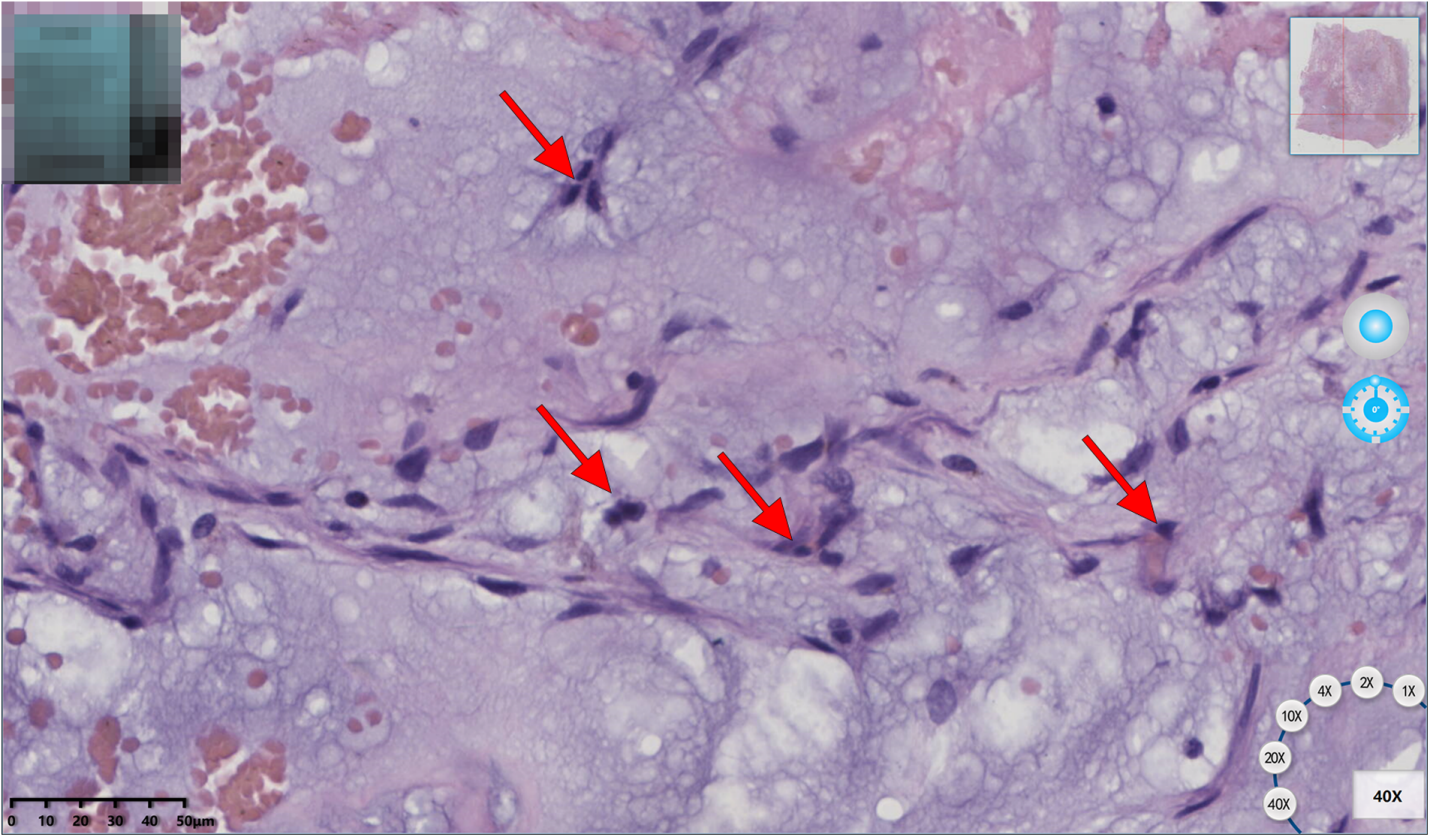
Histology found that myxomatous cells were irregular in shape and sparsely dispersed in the interstitial spaces (red arrow).

The patient underwent a resection of the mass (measuring 110 × 51 × 43 mm, R0 as assessed by histology) (Figure 2) with concomitant tricuspid annuloplasty under cardiopulmonary bypass. The biopsy showed a hemorrhagic and colloidal appearance, and a diagnosis of RV myxoma was made. During the procedure, the heart was exposed through a median sternotomy for gaining a better surgical view of the resection area and cardiac mass removal. A right atriotomy was performed parallel to the atrioventricular groove to fully reveal the myxoma, which was completely resected along its pedicle without the occurrence of any massive intraoperative hemorrhage. An intraoperative investigation did not reveal any right atrium mass. Then, Kay tricuspid annuloplasty was performed to ensure normal hemodynamics of the right heart. The postoperative recovery course was uneventful, and the patient was discharged without any complications. A histological study later confirmed that the myxomatous cells were irregular in shape and sparsely dispersed in the interstitial space (Figure 3). The differential diagnosis of cardiac myxoma should include other benign cardiac tumors such as lipoma, papillary fibroelastoma, and rhabdomyoma, together constituting a similar proportion of benign cardiac tumors. At the 15-month postoperative follow-up, the patient resumed normal activities with a complete resolution of his symptoms, and TTE showed the competence of tricuspid and pulmonary valves with satisfactory hemodynamic control. The patient was instructed to receive periodic echocardiographic follow-up examinations. If this patient had been left to progress without any medical interventions, the myxoma would have continued to simulate the right ventricular outflow tract and even caused pulmonic valve obstruction. In addition, if myxoma detachment had occurred, the right-sided myxomatous emboli might have obstructed the pulmonary arteries and caused pulmonary hypertension and even death from acute obstruction.

## Discussion

In this study, we reported the case of a 53-year-old male patient with a giant ventricular cardiac myxoma, who was admitted to our hospital with a 3-day history of chest tightness and shortness of breath after physical activity. The local hospital, which was the patient’s initial point of contact, performed TTE and found a space-occupying lesion in the right ventricle and its outflow tract, which was further confirmed by TTE, CCTA, and histological examination after admission to our hospital.

Cardiac myxomas usually arise solitarily in the left atrium (75%), right atrium (10%–20%), and heart ventricle (5%), and most have a size of a few centimeters (5, 6). The occurrence of myxoma is approximately 3 per 2 million population, and a right ventricular myxoma with concomitant tricuspid regurgitation is even rarer (7). To the best of our knowledge, only a few myxomas have a size of >110 mm in diameter, let alone locating at a particular anatomical site, the right ventricle, at the same time (8). Compared with other reported cases of cardiac myxoma, this case draws special attention because of the giant size and anatomical location of the myxoma. Although the myxoma protruded into the RV outflow tract and proximal main pulmonary artery during systole, the patient presented with reduced physical performance and signs of anomalous venous drainage other than shock or aborted sudden cardiac death, indicating that the clinical presentation was not severe. Considering the benign characteristics of myxoma, pericardial fluid analysis and positron emission tomography were not performed. Carney complex was ruled out because of the nature of the patient's presentation; Carney complex is a genetic disorder characterized by a combination of cardiac and cutaneous myxomas, endocrine hyperfunction, and distinctive pigmented lesions of the skin and mucosal surfaces (9). As for surgical management, resection is the only effective therapeutic option for ventricular myxoma and should not be delayed because death from obstruction may occur in patients awaiting an operation. Ventricular myxomas are usually approached through the atrioventricular valve for exposure and resection. The approach through a direct incision into the ventricle is not preferred. The operation should be performed without full-thickness excision of the ventricular wall, given that the recurrence rate of cardiac myxoma is approximately 5% and may be observed even months or years after surgery (10). In addition, long-term follow-up with TTE is needed in all patients in order to improve their quality of life and decrease morbidity and mortality rates (11). We are aware that our case report might have some limitations. First, the patient did not undergo cardiac magnetic resonance imaging, which involves a more thorough examination of the soft tissue and has a higher temporal resolution (12). In addition, it remains difficult to assess the long-term survival rates because of an inadequate follow-up time of 15 months.

In our case, the characteristics of the patient were middle age, a giant right ventricular myxoma, and RV outflow tract obstruction, which may provide important insights for cardiologists and cardiac surgeons. In addition, surgical resection and tricuspid annuloplasty showed a good response in our patient. Further follow-up and more clinical experience are required for providing better treatment for those with unique myxomas.

## Data Availability

The original contributions presented in the study are included in the article/**Supplementary Material**; further inquiries can be directed to the corresponding author.

## References

[1] Reynen K. Frequency of primary tumors of the heart. Am J Cardiol. (1996) 77:107. 10.1016/S0002-9149(97)89149-7 8540447

[2] Poterucha TJ Kochav J O’Connor DS Rosner GF. Cardiac tumors: clinical presentation, diagnosis, and management. Curr Treat Options Oncol. (2019) 20:66. 10.1007/s11864-019-0662-1 31250250

[3] Pinede L Duhaut P Loire R. Clinical presentation of left atrial cardiac myxoma. A series of 112 consecutive cases. Medicine (Baltimore). (2001) 80:159–72. 10.1097/00005792-200105000-00002 11388092

[4] Samanidis G Khoury M Balanika M Perrea DN. Current challenges in the diagnosis and treatment of cardiac myxoma. Kardiologia polska. (2020) 78(4):269–77. 10.33963/KP.15254 32207702

[5] Jaravaza DR Lalla U Zaharie SD de Jager LJ. Unusual presentation of atrial myxoma: a case report and review of the literature. Am J Med Case Rep. (2021) 22:e931437. 10.12659/AJCR.931437 PMC8105743 33939684

[6] Li Y Li X Wang X Chen L. Biatrial myxoma floating like a butterfly: a case report and review of the literature. Medicine (Baltimore). (2018) 97:e9558. 10.1097/MD.0000000000009558 29504977 PMC5779746

[7] Kacar P Pavsic N Bervar M Strazar ZD Zadnik V Jelenc M Cardiac myxoma: single tertiary centre experience. Radiol Oncol. (2022) 56:535–40. 10.2478/raon-2022-0041 36259335 PMC9784375

[8] Wang H Li Q Xue M Zhao P Cui J. Cardiac myxoma: a rare case series of 3 patients and a literature review. J Ultrasaound Med. (2017) 36:2361–6. 10.1002/jum.14264 28556391

[9] Correa R Salpea P Stratakis CA. Carney complex: an update. Eur J Endocrinol. (2015) 173:M85–97. 10.1530/EJE-15-0209 26130139 PMC4553126

[10] Pitsava G Zhu C Sundaram R Mills JL Stratakis CA. Predicting the risk of cardiac myxoma in carney complex. Genetics in medicine: official journal of the American College of Medical Genetics. (2021) 23(1):80–5. 10.1038/s41436-020-00956-3 32893266 PMC7796922

[11] Gaszewska-Żurek E Zurek P Wilczyński M Krzych Ł Bachowski R Jasiński M Cardiac myxoma – clinical presentation and long-term post-operative follow-up. Kardiol Pol. (2011) 69:329–34. PMID: 21523664

[12] Kassop D Donovan MS Cheezum MK Nguyen BT Gambill NB Blankstein R Cardiac masses on cardiac CT: a review. Curr Cardiovasc Imaging Rep. (2014) 7:9281. 10.1007/s12410-014-9281-1 25018846 PMC4090749

