# Supplementary figures and images for: Case report: Resection of a giant right ventricular myxoma
Source: Front Surg. 2023 Feb 27;10:1140016. doi: 10.3389/fsurg.2023.1140016 (PMC10009172; doi:10.3389/fsurg.2023.1140016)

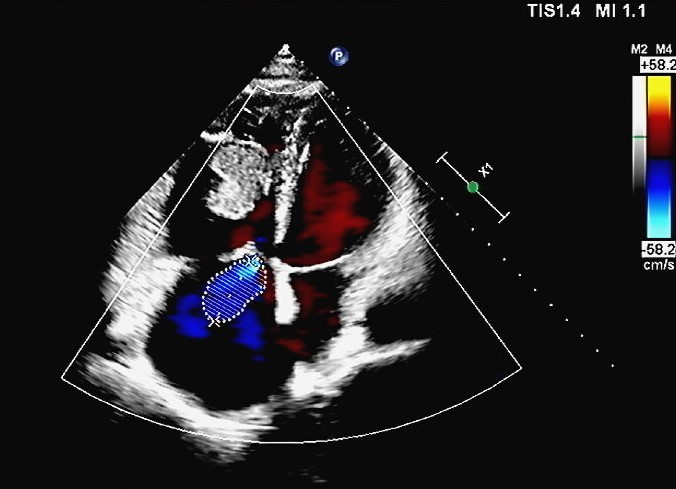

Supplement: Supplementary file 2 [file Image1.jpeg]

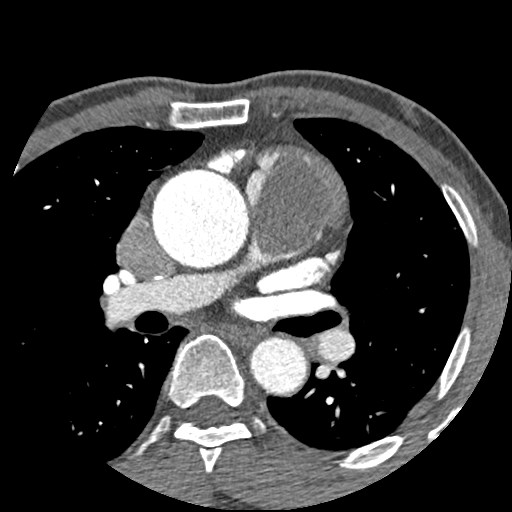

Supplement: Supplementary file 3 [file Image2.jpeg]
